# Supplementary material for: Risk Factors for Low Quality of Life among Women Using Different Types of Contraceptives in Saudi Arabia: A Questionnaire-Based Study
Source: Behav Sci (Basel). 2024 Sep 17;14(9):829. doi: 10.3390/bs14090829 (PMC11429329; doi:10.3390/bs14090829)
Supplement: Supplementary file 1 [file behavsci-14-00829-s001.zip › behavsci-3120438-supplementary.pdf]

**Table S1:** Items modified from the SEC-QOL questionnaire.

| Code | Description of variable |
|---|---|
| Ph_1 | I have menstrual pain (pain in the lumbar area and abdomen) a few days before my period starts. |
| Ph_2 | I feel discomfort in the ovary area during my period. |
| Ph_3 | I feel pain in my legs before and during menstruation. |
| Ph_4 | My weight increases and I become edematous. |
| Ph_5 | I feel I want to eat sweets and carbohydrates. |
| Sex_1 | I feel worried that sexual acts affect the efficacy of the method I use. |
| Sex_2 | I feel safe and comfortable during sexual relations because I use contraceptives. |
| Sex_3 | Sexual desire decreases when I use contraceptives. |
| Sex_4 | Using a contraceptive method affects the intensity and length of the menstrual period, which negatively affects my sexual relations. |
| Psy_1 | I feel depressed. |
| Psy_2 | I feel worried that I have hormonal changes. |
| Psy_3 | I get angry quickly. |
| Psy_4 | I feel I cannot concentrate at work. |
| Psy_5 | I feel nervous and impatient. |
| Soc_1 | I do not have the energy to do things that need effort. |
| Soc_2 | I feel that contraceptives interfere with my work performance. |
| Soc_3 | I prefer quiet social activities. |
| Soc_4 | I avoid social and physical activities that require energy. |
| Br_1 | I feel my breasts are hard and sluggish. |
| Br_2 | I feel that my breasts have increased in size. |

Ph=Physiological domain, Sex= Sexual domain, Psy=Psychological domain, Soc= Social activity domain, Br=Breast domain
